# Dietary intake of methyl-donor nutrients is associated with frailty and partially mediated by oxidative stress in U.S. middle-aged and older adults

**DOI:** 10.1016/j.jnha.2026.101025

**Published:** 2026-11-04

**Authors:** Galya Bigman, Hira Shakoor, Amber S. Kleckner, Alice S. Ryan, Sausan M. Jaber, John D. Sorkin

**Affiliations:** a Department of Epidemiology and Public Health, Division of Gerontology, University of Maryland School of Medicine, Baltimore, MD 21201, United States; b Department of Nutrition and Dietetics, Faculty of Health Sciences, Istanbul Gelisim University, 34310 Istanbul, Turkey; c Department of Nutrition and Exercise Physiology, Elson S. Floyd College of Medicine, Washington State University, Spokane, WA 99202, United States; d Department of Pain and Translational Symptom Science, University of Maryland School of Nursing, Baltimore, MD, United States; e Geriatric Research, Education, and Clinical Center (GRECC), Veterans Affairs Maryland Health Care System, Baltimore, MD 21201, United States; f Division of Gerontology, Geriatrics, and Palliative Medicine, Department of Medicine, University of Maryland School of Medicine, Baltimore, MD 21201, United States

**Keywords:** Frailty, Methyl-donor nutrients, One-carbon metabolism, Oxidative stress, Healthy eating index, Dietary quality, NHANES, Aging, Frailty index, Mediation analysis

## Abstract

**Background:**

Frailty reflects declining physiological reserve and is a major barrier to healthy longevity. Methyl-donor nutrients, which serve as substrates and cofactors in one-carbon metabolism, are involved in several biological processes relevant to aging, including the regulation of oxidative stress. However, their association with frailty and the potential role of oxidative stress in this relationship remain unclear.

**Objective:**

To examine the association between a composite Methyl-Donor Nutrient Quality Index (MNQI) and frailty and to evaluate whether oxidative stress may contribute to this association in middle-aged and older adults.

**Methods:**

Cross-sectional data from NHANES 2005–2018 were analyzed among 13,528 adults aged ≥40 years. Frailty was assessed using a 53-item deficit accumulation frailty index categorized as robust, prefrail, or frail. The MNQI was calculated from seven methyl-donor and cofactor nutrients. The oxidative stress index (OSI) was derived from gamma-glutamyl transferase, uric acid, albumin, and bilirubin. Survey-weighted multinomial logistic regression examined MNQI and OSI associations with frailty, and exploratory mediation analyses evaluated the indirect effect through OSI.

**Results:**

Higher MNQI was associated with 33% lower odds of frailty (OR = 0.67; 95% CI: 0.52–0.88; P = 0.004), with a significant dose–response relationship. Higher OSI was associated with 113% greater odds of frailty (OR = 2.13; 95% CI: 1.92–2.36; P < 0.001). Exploratory mediation analyses indicated a significant indirect effect through oxidative stress, accounting for approximately 29% of the MNQI–frailty association.

**Conclusions:**

Higher dietary intake of methyl-donor nutrients was associated with lower odds of frailty and may support physiological processes relevant to healthy longevity, with oxidative stress potentially representing one pathway underlying this association. Longitudinal studies and randomized trials are needed to confirm these relationships and clarify the underlying mechanisms.

## 1 Introduction

Frailty is a common age-related clinical syndrome characterized by reduced physiological reserve and cumulative dysregulation across multiple biological systems [[1], [2], [3], [4]]. It reflects declining intrinsic capacity across physical, cognitive, psychological, and functional domains, rendering individuals increasingly vulnerable to stressors such as infection, injury, and psychosocial challenges [[5], [6], [7]]. As such, frailty represents an important barrier to healthy aging and longevity. Although frailty often coexists with multimorbidity and disability, it is a distinct clinical entity with specific measures. One of the most widely used approaches to quantify frailty is the Frailty Index (FI), which operationalizes frailty by measuring the accumulation of health deficits across multiple domains [8,9], thereby capturing its multidimensional and dynamic nature.

Nutrition is a key modifiable determinant of healthy aging. Numerous studies have shown that healthy dietary patterns, including the Mediterranean diet, DASH, and the Healthy Eating Index, are associated with a lower risk of frailty [[10], [11], [12], [13], [14], [15], [16], [17], [18]], potentially through mechanisms involving reduced inflammation, attenuation of oxidative stress [19,20], and preservation of muscle mass and function [10,11]. While these dietary patterns provide important measures of overall diet quality, examining specific nutrients may provide additional insight into metabolic pathways relevant to frailty. Inadequate intake of specific nutrients may offer additional mechanistic insight by disrupting biological processes underlying functional domains relevant to frailty. For example, inadequate intake of B vitamins impairs one-carbon metabolism and mitochondrial function [21], while insufficient protein intake compromises muscle mass and physical performance [22], both of which are central to frailty pathophysiology [10,14,[23], [24], [25], [26], [27],^28^]. Nevertheless, the specific nutrient pathways through which diet confers protection against frailty remain incompletely understood.

Increasing attention in aging research has been directed toward nutrients involved in one-carbon metabolism (OCM), including methionine, amino acid precursors, B-complex vitamins, choline, and zinc. These nutrients provide methyl-donor substrates or act as cofactors within one-carbon metabolism, supporting processes including DNA methylation, homocysteine metabolism, mitochondrial function, and antioxidant defense [[29], [30], [31], [32]]. Although traditionally studied in maternal and child health, they are increasingly recognized for their relevance to healthy aging and longevity.

Specifically, higher dietary intake of methyl-donor nutrients has been associated with lower phenotypic age acceleration and healthier epigenetic aging profiles [33], neuroprotection and reduced cognitive impairment [34], improved sleep quality and gut microbiota regulation in older adults [35,36], and lower risk of metabolic syndrome and type 2 diabetes [37]. These converging findings suggest that dietary intake of methyl-donor nutrients, primarily through their role in one-carbon metabolism, may support physiological domains closely linked to frailty, including cognitive function, metabolic regulation, and resilience to age-related decline.

Despite this biological rationale, dietary intake of methyl-donor nutrients as a composite measure has not been investigated in relation to frailty, particularly its multidimensional operationalization as captured by the FI. Because these nutrients function interdependently within OCM, examining them in isolation risks obscuring the collective nutritional burden that may drive frailty risk. Evaluating methyl-donor nutrients as a composite dietary construct may therefore provide a biologically meaningful approach for capturing nutritional exposures relevant to frailty through interconnected aging-related pathways.

Consequently, to fill this gap in the aging and nutrition literature, the objective of this study was to examine the association between the Methyl-Donor Nutrient Quality Index (MNQI) and multidimensional frailty, independent of overall diet quality, and to evaluate oxidative stress as a potential mediating pathway in a NHANES sample of U.S. middle-aged and older adults. We hypothesized that higher MNQI would be associated with lower odds of frailty and that oxidative stress would partially account for this association.

## 2 Methods

### 2.1 Study population

This cross-sectional study used data from the National Health and Nutrition Examination Survey (NHANES) 2005–2018. NHANES employs a complex, multistage, stratified probability sampling design to obtain a sample of civilian, noninstitutionalized U.S. population [38,39]. The study protocol was approved by the National Center for Health Statistics Research Ethics Review Board [39]; the present study is a secondary analysis of publicly available, de-identified data and was therefore exempt from institutional review board review. All participants provided written informed consent. Ethical approval was obtained for each survey cycle, including protocols #2005–06, #2011–17, and #2018–01 and their continuations. Analyses were restricted to NHANES cycles from 2005 through 2018 to avoid potential disruptions in dietary patterns and health status during the COVID-19 pandemic and due to the absence of key variables in later cycles. Adults aged ≥40 years were included. Participants with insufficient FI item completion (<80%) or missing data on dietary intake, biomarkers, or required covariates were excluded (Supplementary Fig. S1).

### 2.2 Frailty assessment

Frailty was assessed using a frailty index (FI) constructed according to the deficit accumulation approach [8,9,40], a well-established and validated method, originally developed by Rockwood and colleagues, that considers the accumulation of age-related health deficits displayed by an individual and has been widely applied in epidemiological research. The FI is calculated as the number of deficits present divided by the total number of potential health deficits considered. Deficits were selected from NHANES variables to represent multiple physiological and functional domains and to meet standard FI criteria [8,9,40]. A total of 53 deficits were grouped into five domains: cognition (1 item), functional limitations (20 items), depressive symptoms (7 items), comorbidities (13 items), and healthcare utilization, self-rated health, and anthropometric and laboratory measures (12 items). Functional limitation items used ordered response categories coded as 0, 0.33, 0.66, and 1, where 0 indicated no deficit and 1 indicated the maximum deficit; depressive symptoms were coded similarly, on a scale from 0 to 1. Binary variables were coded as 0 (absent) or 1 (present), with selected variables assigned intermediate values to reflect graded responses. Laboratory values were coded as 0 (within reference range) or 1 (outside reference range). The FI score was calculated as the sum of deficits divided by the number of non-missing items, with missing items not coded as 0, yielding a value ranging from 0 to 1, with higher values indicating a greater degree of frailty; participants with data available for at least 80% of items (≥43/53) were included in the analysis. FI categories were defined as robust (FI < 0.10), prefrail (FI 0.10–0.24), and frail (FI ≥ 0.25). Full details of the FI variables and scoring are provided in Supplementary Table S1.

### 2.3 Dietary assessment

Dietary intake was assessed using two 24-h dietary recalls collected with the USDA Automated Multiple-Pass Method (AMPM) [38]. The first recall was conducted in person at the Mobile Examination Center; the second was obtained by telephone 3–10 days later. Consistent with standard NHANES methodology, mean intake from two recalls was used when available; otherwise, data from a single recall were used [38].

### 2.4 Methyl-donor nutrient quality index (MNQI)

Seven dietary methyl-donor nutrients were selected a priori based on their established roles in one-carbon metabolism: protein, folate, choline, riboflavin, vitamin B6, vitamin B12, and zinc [21,29,30,33,41]. Protein was included as a surrogate source of dietary methionine because methionine intake is not available in NHANES dietary nutrient files. These nutrients were selected to represent both methyl-group donors and essential enzymatic cofactors involved in one-carbon metabolism. A composite Methyl-Donor Nutrient Quality Index (MNQI) was constructed to reflect overall adequacy of these nutrients [33,41,42]. For each nutrient, intake was compared with age- and sex-specific recommended levels; adequacy was defined as intake at or above two-thirds of the recommended level and, where applicable, not exceeding the tolerable upper intake level (UL). No UL was applied for riboflavin or vitamin B12 due to the absence of established limits. Protein adequacy was defined as 0.8–2.0 g/kg/day using measured body weight. Each nutrient was assigned a score of 0 if intake fell outside the adequacy range, or a fixed positive score if intake was within range. Protein, folate, and choline were assigned 2 points each because they serve as primary methyl-group donors or major substrates within one-carbon metabolism, whereas riboflavin, vitamin B6, vitamin B12, and zinc were assigned 1 point each because they function primarily as enzymatic cofactors supporting these metabolic pathways [21,29,30]. Scores were summed to generate a total MNQI ranging from 0 to 10, with higher scores indicating greater methyl-donor nutrient adequacy [[33], [34], [35],^41^,^42^]. For analysis, MNQI was categorized into tertiles. Nutrient-specific cut-points and scoring criteria are provided in Supplementary Table S2.

### 2.5 Oxidative stress index (OSI)

The oxidative stress index (OSI), a composite measure of pro-oxidant and antioxidant balance, was derived from four serum biomarkers: gamma-glutamyl transferase (GGT) and uric acid (pro-oxidant markers), and total bilirubin and albumin (endogenous antioxidants), consistent with prior NHANES-based studies [43,44]. All four were assayed on the same platform (Beckman Coulter UniCel DxC800): uric acid and bilirubin via timed-endpoint methods (bilirubin specifically via the diazo [Jendrassik-Grof] method), albumin via a bichromatic digital endpoint assay, and GGT via an enzymatic rate method. Assay protocols and quality-control documentation are publicly available, and all measurements followed standardized NHANES laboratory procedures [39]. Because GGT showed marked right skewness, it was log-transformed prior to standardization; the remaining biomarkers were retained on their original scale. Each biomarker was then standardized to a z-score, and because bilirubin and albumin are inversely associated with oxidative stress, their z-scores were multiplied by −1 [43,44]:

OSI = [z(ln GGT) + z(Uric Acid) − z(Total Bilirubin) − z(Albumin)] / 4

The OSI thus represents the mean of the four standardized values, with higher values indicating greater oxidative stress. The index was then re-standardized to a mean of 0 and SD of 1 to facilitate interpretation of regression coefficients as effects per 1-SD increase in oxidative stress.

### 2.6 Covariates

Potential covariates were identified a priori based on biological plausibility and prior literature and included demographic, socioeconomic, lifestyle, and dietary factors. Demographic variables included age (continuous), sex (male/female), race/ethnicity (non-Hispanic White, Hispanic, non-Hispanic Black, and non-Hispanic Asian/other), education level (less than high school, high school or equivalent, above high school), and marital status (married or living with partner vs. living alone). Lifestyle factors included smoking status (never, former, current), alcohol consumption (never, former, current), body mass index (BMI, kg/m²; continuous), and overall diet quality assessed using the Healthy Eating Index-2020 (HEI) [45,46]. Total energy intake (kcal/day) and HEI were derived from the mean of two 24-h dietary recalls. Chronic disease burden was operationalized as a categorical variable reflecting the number of self-reported physician-diagnosed conditions, including cardiovascular disease, cancer, chronic respiratory disease, liver disease, diabetes, and kidney disease. Chronic disease burden was categorized as 0, 1, or ≥2 conditions.

### 2.7 Statistical analysis

All analyses accounted for the complex NHANES survey design by incorporating sampling weights, strata (SDMVSTRA), and primary sampling units (SDMVPSU) to produce nationally representative estimates [38,39]. The dietary sample weight (WTDR2D) was divided by 7 to account for the seven combined survey cycles, consistent with NHANES analytic guidelines. Continuous variables are presented as survey-weighted means ± SD; categorical variables are presented as weighted percentages. Differences across frailty categories were assessed using survey-weighted linear regression for continuous variables and survey-weighted chi-square tests for categorical variables.

Associations between MNQI and frailty and between oxidative stress biomarkers, OSI and frailty were each examined using survey-weighted multinomial logistic regression, with the robust group as the reference category. Three models were specified: Model 1 was unadjusted; Model 2 adjusted for age, sex, race/ethnicity, education, and marital status; and Model 3 further adjusted for smoking status, alcohol use, HEI, total energy intake, and BMI. Individual oxidative stress biomarkers were modeled as continuous variables in their original units, with GGT log-transformed because of its skewed distribution. The composite OSI was standardized (mean = 0, SD = 1), and its association with frailty was expressed per 1-SD increase in oxidative stress. Results are reported as odds ratios (ORs) with 95% confidence intervals (CIs).

Candidate covariates were subsequently evaluated as potential confounders based on: (1) P < 0.10 in bivariate analyses with frailty status; (2) clinical relevance based on existing literature; and (3) a change-in-estimate criterion, retaining variables that changed the main exposure OR by ≥10% for either frailty category relative to the robust reference group.

Exploratory mediation analysis was conducted using a counterfactual framework to estimate the average causal mediation effect (ACME) and average direct effect (ADE), implemented with the mediation package (version 4.5.1) in R. Separate analyses were conducted for the prefrail vs. robust and frail vs. robust contrasts, using binary logistic regression for the outcome model and linear regression for the mediator model. MNQI tertiles served as the exposure, OSI as the mediator, and frailty status as the outcome, adjusted for the same covariates as Model 3. Sampling weights were included as a continuous covariate to partially account for the complex survey design; full probability weighting was not applied due to software constraints. Statistical inference was based on nonparametric bootstrap confidence intervals derived from 2000 replications (seed = 2026). ACME, ADE, total effects, and the estimated proportion mediated are reported.

Sensitivity analyses were conducted to assess the robustness of the findings. First, to assess whether the association between MNQI and frailty differed according to overall diet quality, we tested the interaction between MNQI tertiles and HEI-2020 tertiles. Second, to assess potential selection bias, characteristics of participants with complete data were compared with those of participants missing data on key study variables. Finally, to assess the potential influence of the lower age threshold used in the primary analysis, we repeated the analysis among adults aged ≥60 years using survey-domain estimation to preserve the complex NHANES survey design. The same sequence of multinomial logistic regression models used in the primary analysis was applied to this age-restricted sample.

Statistical significance was defined as a two-sided P < 0.05. All statistical analyses were conducted using Stata version 19.5 (StataCorp LLC, College Station, TX), with mediation analyses performed in R version 4.5.1.

## 3 Results

### 3.1 Characteristics of the study participants

Among 13,528 U.S. adults aged ≥40 years, 23.6% were classified as frail, 56.7% as prefrail, and 19.7% as robust (Table 1). The relatively high prevalence of frailty should be interpreted in the context of NHANES survey design, whereby participants reporting no limitations in work ability, mobility, or cognition were not administered the detailed physical functioning module by design, and were therefore more likely to have insufficient data for frailty index construction and be excluded from the analytic sample (Fig. S1, Supplementary Table S1).

**Table 1:** Weighted characteristics of U.S. adults by frailty status.

Characteristic	Overall	*Robust*	*Prefrail*	*Frail*	P value
	N = 13,528	N = 2407	N = 7449	N = 3672	
**Age,**^1^**y,** mean ± SD	64.7 ± 10.5	63.9 ± 8.6	65.4 ± 10.3	63.6 ± 12.6	<0.001
**Sex, n (%)**					<0.001
Male	6686 (45.9)	1417 (54.0)	3719 (46.5)	1550 (37.7)	
Female	6842 (54.1)	990 (46.0)	3730 (53.5)	2122 (62.3)	
**Race/ethnicity,** n (%)					<0.001
Non-Hispanic White	6793 (76.1)	1246 (81.9)	3813 (77.5)	1734 (68.1)	
Hispanic	2914 (8.6)	570 (7.0)	1545 (8.0)	799 (11.2)	
Non-Hispanic Black	2823 (9.7)	357 (5.6)	1571 (9.3)	895 (14.2)	
Asian/Other	998 (5.6)	234 (5.5)	520 (5.3)	244 (6.5)	
**Education level,** n (%)					<0.001
Less than high school	3979 (18.3)	553 (11.8)	2041 (16.7)	1385 (27.6)	
High school/equivalent	3291 (25.8)	529 (23.6)	1850 (25.2)	912 (29.2)	
Above high school	6258 (55.9)	1325 (64.6)	3558 (58.1)	1375 (43.2)	
**Marital status,** n (%)					<0.001
Married/living with partner	7796 (63.3)	1625 (73.1)	4395 (64.3)	1776 (52.5)	
Living alone	5732 (36.7)	782 (26.9)	3054 (35.7)	1896 (47.5)	
**Smoking status,** n (%)					<0.001
Never	6332 (47.1)	1257 (53.7)	3588 (48.5)	1487 (38.4)	
Former	4787 (35.6)	774 (32.1)	2698 (37.1)	1315 (34.8)	
Current	2400 (17.3)	374 (14.2)	1157 (14.4)	869 (26.8)	
**Alcohol use,**^2^ n (%)					<0.001
Never	2068 (12.3)	336 (11.3)	1122 (12.4)	610 (13.1)	
Former	3827 (25.0)	444 (14.5)	1989 (24.6)	1394 (34.4)	
Current	7470 (62.7)	1572 (74.2)	4178 (63.0)	1720 (52.5)	
**Diet quality (HEI),** mean ± SD	55.5 ± 13.3	58.0 ± 13.0	55.9 ± 13.1	52.6 ± 13.3	<0.001
**BMI,**^3^**kg/m²,** mean ± SD	29.8 ± 6.9	26.7 ± 4.4	29.7 ± 6.4	32.7 ± 8.7	<0.001
**Total energy intake, kcal/day,** mean ± SD	1920 ± 733	2006 ± 699	1936 ± 716	1808 ± 786	<0.001
**Chronic disease burden,** n (%)					<0.001
None	3061 (22.2)	1498 (59.6)	1406 (17.1)	157 (4.3)	
One condition	4408 (34.1)	781 (34.7)	2894 (38.8)	733 (22.1)	
Two or more conditions	6059 (43.7)	128 (5.7)	3149 (44.1)	2782 (73.6)	
**MNQI,**^4^**n (%)**					<0.001
T1 (score 0–7)	5418 (35.3)	813 (27.9)	2820 (33.5)	1785 (46.0)	
T2 (score 8)	4218 (32.5)	819 (35.4)	2426 (34.2)	973 (26.0)	
T3 (score 9–10)	3892 (32.2)	775 (36.7)	2203 (32.3)	914 (28.1)	
**Methyl-Donor Nutrients**, mean ± SD					
Protein, g/day	74.9 ± 31.4	79.8 ± 32.4	75.7 ± 31.0	69.2 ± 30.9	<0.001
Folate, μg/day	378 ± 194	411 ± 212	384 ± 194	340 ± 176	<0.001
Choline, mg/day	316 ± 152	334 ± 151	318 ± 152	296 ± 151	<0.001
Riboflavin, mg/day	2.08 ± 0.97	2.20 ± 1.03	2.09 ± 0.96	1.94 ± 0.95	<0.001
Vitamin B6, mg/day	1.93 ± 1.12	2.06 ± 1.08	1.95 ± 1.10	1.79 ± 1.18	<0.001
Vitamin B12, μg/day	5.08 ± 5.87	5.29 ± 5.61	5.13 ± 5.76	4.78 ± 6.23	0.094
Zinc, mg/day	10.9 ± 6.58	11.6 ± 6.38	11.1 ± 7.01	10.0 ± 5.70	<0.001
**Oxidative Stress Biomarkers,** mean ± SD					
Uric acid,^6^ mg/dL, mean ± SD	5.61 ± 1.50	5.37 ± 1.21	5.60 ± 1.38	5.83 ± 1.73	<0.001
GGT,^7^ U/L, mean ± SD	31.0 ± 46.4	24.0 ± 19.2	30.1 ± 43.3	38.8 ± 60.6	<0.001
Bilirubin,^8^ mg/dL, mean ± SD	0.65 ± 0.30	0.73 ± 0.29	0.65 ± 0.28	0.58 ± 0.30	<0.001
Albumin^9^, g/dL	4.17 ± 0.32	4.27 ± 0.26	4.20 ± 0.30	4.06 ± 0.40	<0.001
**Oxidative stress index (OSI),**^5^ mean ± SD	−0.09 ± 0.97	−0.53 ± 0.83	−0.14 ± 0.99	0.38 ± 1.09	<0.001

Values are survey-weighted means ± SD or n (weighted %). P values were derived from survey-weighted linear regression for continuous variables and survey-weighted χ² tests for categorical variables. All analyses accounted for the complex survey design and sample weights.

1 Age is presented in years.

2 There were 163 missing values for alcohol use.

3 BMI, body mass index.

4 MNQI, Methyl-Donor Nutrient Quality Index (range 0–10; higher scores indicate greater methyl-donor nutrient adequacy); tertiles defined as T1 (0–7), T2 (8), and T3 (9–10).

5 Oxidative stress index (OSI) represents a standardized composite z-score combining uric acid, GGT [log-transformed], bilirubin, and albumin, with higher values indicating greater oxidative stress.

6 Uric acid: pro-oxidant marker; higher values indicate greater oxidative stress.

7 GGT, γ-glutamyltransferase: pro-oxidant marker; higher values indicate greater oxidative stress.

8 Bilirubin: antioxidant marker; lower values indicate greater oxidative stress.

9 Albumin: antioxidant marker; lower values indicate greater oxidative stress.

Frail individuals were more likely to be women, belong to racial/ethnic minority groups, have lower educational attainment, live alone, be current smokers, and report former alcohol use compared with robust individuals. Robust individuals were more likely to report current alcohol consumption and be married or living with a partner. Frail individuals also exhibited less favorable nutritional and metabolic profiles, including higher BMI and oxidative stress levels, lower diet quality, and a higher proportion in the lowest MNQI tertile, whereas robust individuals had the highest proportion in the highest MNQI tertile. All characteristics differed significantly across frailty categories in survey-weighted analyses (P < 0.001).

Participants excluded due to missing data differed significantly from those included in the analytic sample (Supplementary Table S3). Excluded individuals had higher energy intake, higher MNQI, lower oxidative stress, and slightly lower BMI, and were more likely to be never smokers and married or partnered but less likely to be non-Hispanic White (all P < 0.001).

### 3.2 Association between MNQI and frailty

In survey-weighted multinomial logistic regression analyses (Table 2), higher MNQI was inversely associated with frailty across all models. Associations with prefrailty were modest and attenuated after adjustment; in the fully adjusted model (Model 3), neither T2 nor T3 was significantly associated with prefrailty, although the trend was borderline significant (P for trend = 0.050). For frailty, inverse associations were stronger and persisted across all models; in Model 3, individuals in T2 and T3 had 23% and 33% lower odds of frailty compared with T1, respectively, with a significant dose–response relationship (P for trend = 0.004). Restricted cubic spline analysis showed no significant departure from linearity for either prefrailty or frailty (P for nonlinearity >0.05; Fig. 1).

**Table 2:** Survey-weighted association between methyl-donor nutrient quality and frailty.

	Model 1		Model 2		Model 3	
MNQI	OR (95% CI)	P value	OR (95% CI)	P value	OR (95% CI)	P value
**Prefrail vs. Robust**						
T2 vs T1	0.83 (0.69–1.00)	0.038	0.91 (0.76–1.08)	0.273	1.01 (0.83–1.23)	0.908
T3 vs T1	0.75 (0.61–0.92)	0.007	0.84 (0.68–1.03)	0.093	0.82 (0.65–1.04)	0.095
P for trend	0.007		0.070		0.050	
**Frail vs. Robust**						
T2 vs T1	0.46 (0.36–0.58)	<0.001	0.58 (0.46–0.73)	<0.001	0.77 (0.60–0.98)	0.037
T3 vs T1	0.45 (0.37–0.56)	<0.001	0.57 (0.46–0.70)	<0.001	0.67 (0.52–0.88)	0.004
P for trend	<0.001		<0.001		0.004	

Survey-weighted multinomial logistic regression. Robust was the reference category.

Model 1: Unadjusted.

Model 2: Adjusted for age, sex, race/ethnicity, education level, and marital status.

Model 3: Adjusted for the variables in Model 2 + smoking status, alcohol use, energy intake, diet quality (HEI), and BMI.

MNQI, Methyl-Donor Nutrient Quality Index; OR, odds ratio; HEI, Healthy Eating Index; BMI, body mass index.

**Fig. 1:**
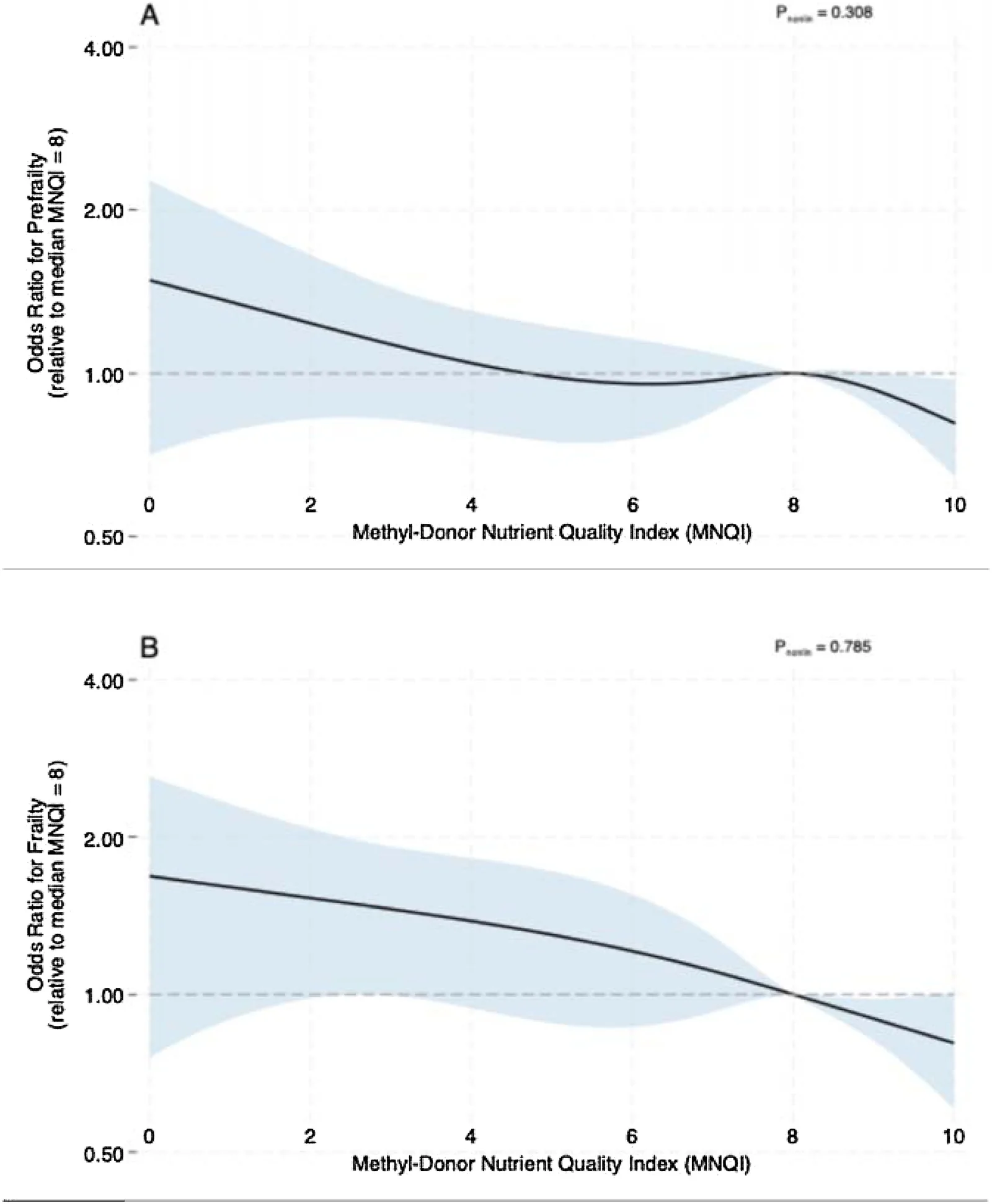
Restricted cubic spline dose-response relationships between the Methyl-Donor Nutrient Quality Index (MNQI) and frailty status. (A) Prefrailty versus robust; (B) frailty versus robust. Odds ratios are expressed relative to the median MNQI (=8), adjusted for Model 3 covariates. Shaded areas = 95% CI. Dashed line indicates OR = 1.0. The associations did not significantly deviate from linearity (Pnonlinearity > 0.05).

### 3.3 Association between oxidative stress and frailty

In models examining individual oxidative stress biomarkers, the two pro-oxidant markers (GGT, uric acid) were positively associated with higher odds of prefrailty and frailty, while the two antioxidant markers (bilirubin, albumin) were inversely associated, consistent with their hypothesized roles (Table 3). These associations were attenuated but remained significant across all models, with the exception of uric acid's association with prefrailty, which was attenuated to the null after adjustment for lifestyle factors **(Model 3).** Higher OSI was independently associated with increased odds of both prefrailty and frailty across all models (Table 3). Associations were attenuated but remained highly significant in the fully adjusted model (Model 3).

**Table 3:** Survey-weighted association between oxidative stress biomarkers, oxidative stress index and frailty.

	Model 1		Model 2		Model 3	
	OR (95% CI)	P value	OR (95% CI)	P value	OR (95% CI)	P value
**Prefrail vs. Robust**						
***Oxidative Stress Biomarkers***						
Uric acid^1^, mg/dL	1.12 (1.06–1.19)	<0.001	1.18 (1.11–1.25)	<0.001	1.04 (0.98–1.10)	0.229
GGT^2^, ln(U/L)	1.39 (1.23–1.58)	<0.001	1.55 (1.36–1.77)	<0.001	1.37 (1.20–1.57)	<0.001
Bilirubin^3^, mg/dL	0.44 (0.35–0.54)	<0.001	0.46 (0.37–0.57)	<0.001	0.53 (0.42–0.66)	<0.001
Albumin^4^, g/dL	0.45 (0.35–0.58)	<0.001	0.51 (0.39–0.65)	<0.001	0.75 (0.57–0.98)	0.035
Oxidative stress index^5^ (OSI)	1.64 (1.50–1.79)	<0.001	1.64 (1.51–1.79)	<0.001	1.37 (1.26–1.49)	<0.001
**Frail vs. Robust**						
***Oxidative Stress Biomarkers***						
Uric acid^1^, mg/dL	1.25 (1.19–1.31)	<0.001	1.37 (1.30–1.45)	<0.001	1.12 (1.06–1.19)	<0.001
GGT^2^, ln(U/L)	2.04 (1.80–2.32)	<0.001	2.24 (1.96–2.55)	<0.001	1.91 (1.66–2.20)	<0.001
Bilirubin^3^, mg/dL	0.15 (0.11–0.20)	<0.001	0.19 (0.14–0.26)	<0.001	0.26 (0.19–0.36)	<0.001
Albumin^4^, g/dL	0.11 (0.09–0.15)	<0.001	0.14 (0.11–0.19)	<0.001	0.30 (0.22–0.41)	<0.001
Oxidative stress index^5^ (OSI)	2.91 (2.62–3.23)	<0.001	2.90 (2.61–3.21)	<0.001	2.13 (1.92–2.36)	<0.001

Survey-weighted multinomial logistic regression. Robust was the reference category. Odds ratios reflect the association per 1-unit increase in each biomarker, except for OSI, for which odds ratios reflect a 1-SD increase. GGT was log-transformed because of its skewed distribution.

Model 1: Unadjusted.

Model 2: Adjusted for age, sex, race/ethnicity, education level, and marital status.

Model 3: Adjusted for the variables in Model 2 + smoking status, alcohol use, energy intake, diet quality (HEI), and BMI.

GGT, gamma-glutamyl transferase; OSI, oxidative stress index; BMI, body mass index; HEI, Healthy Eating Index.

### 3.4 Role of oxidative stress in the association between MNQI and frailty

Mediation analyses based on the counterfactual framework examining the role of oxidative stress in the association between MNQI and frailty are presented in Table 4. For the frail vs. robust contrast, the exploratory mediation analysis indicated a significant indirect effect through oxidative stress for both the middle tertile (T2 vs. T1: ACME = −0.010; 95% CI, −0.017 to −0.002; P = 0.013) and the highest tertile (T3 vs. T1: ACME = −0.014; 95% CI, −0.023 to −0.006; P < 0.001), accounting for approximately 29.2% and 28.5% of the total effect, respectively.

**Table 4:** Mediation analysis: role of oxidative stress index in the association between MNQI and frailty.

Outcome / Comparison	ACME	95% CI	P value	Prop. Mediated	ADE	ADE P value	Total Effect P value
**Prefrail vs. Robust**							
T2 vs. T1	−0.001	−0.003, 0.001	0.368	—	0.007	0.541	0.607
T3 vs. T1	−0.001	−0.004, 0.002	0.465	—	−0.012	0.353	0.334
**Frail vs. Robust**							
T2 vs. T1	−0.010	−0.017, −0.002	0.013	29.2%	−0.024	0.095	0.025
T3 vs. T1	−0.014	−0.023, −0.006	<0.001	28.5%	−0.035	0.022	<0.001

ACME, average causal mediation effect; ADE, average direct effect; CI, confidence interval; MNQI, Methyl-Donor Nutrient Quality Index; OSI, oxidative stress index (mediator).

Nonparametric bootstrap with percentile confidence intervals, 2000 simulations (seed = 2026). Robust was the reference category for frailty status; T1 was the reference category for MNQI tertile.

Mediator (OSI) and outcome models adjusted for age, sex, race/ethnicity, education, marital status, smoking status, BMI, overall diet quality (HEI-2020), energy intake, and alcohol use, among participants with WTDR2D_14YR > 0.

Prop. Mediated is reported only when the ACME was statistically significant (P < 0.05); estimates are unstable and not interpretable otherwise.

The total effect of MNQI on frailty was significant for both contrasts (T2: P = 0.025; T3: P < 0.001), while the direct effect remained significant for the T3 contrast (P = 0.022) but did not reach significance for T2 (P = 0.095) (Fig. 2). No significant indirect effect through oxidative stress was observed for the prefrail vs. robust contrast (ACME P = 0.368 and P = 0.465 for T2 and T3, respectively).

**Fig. 2:**
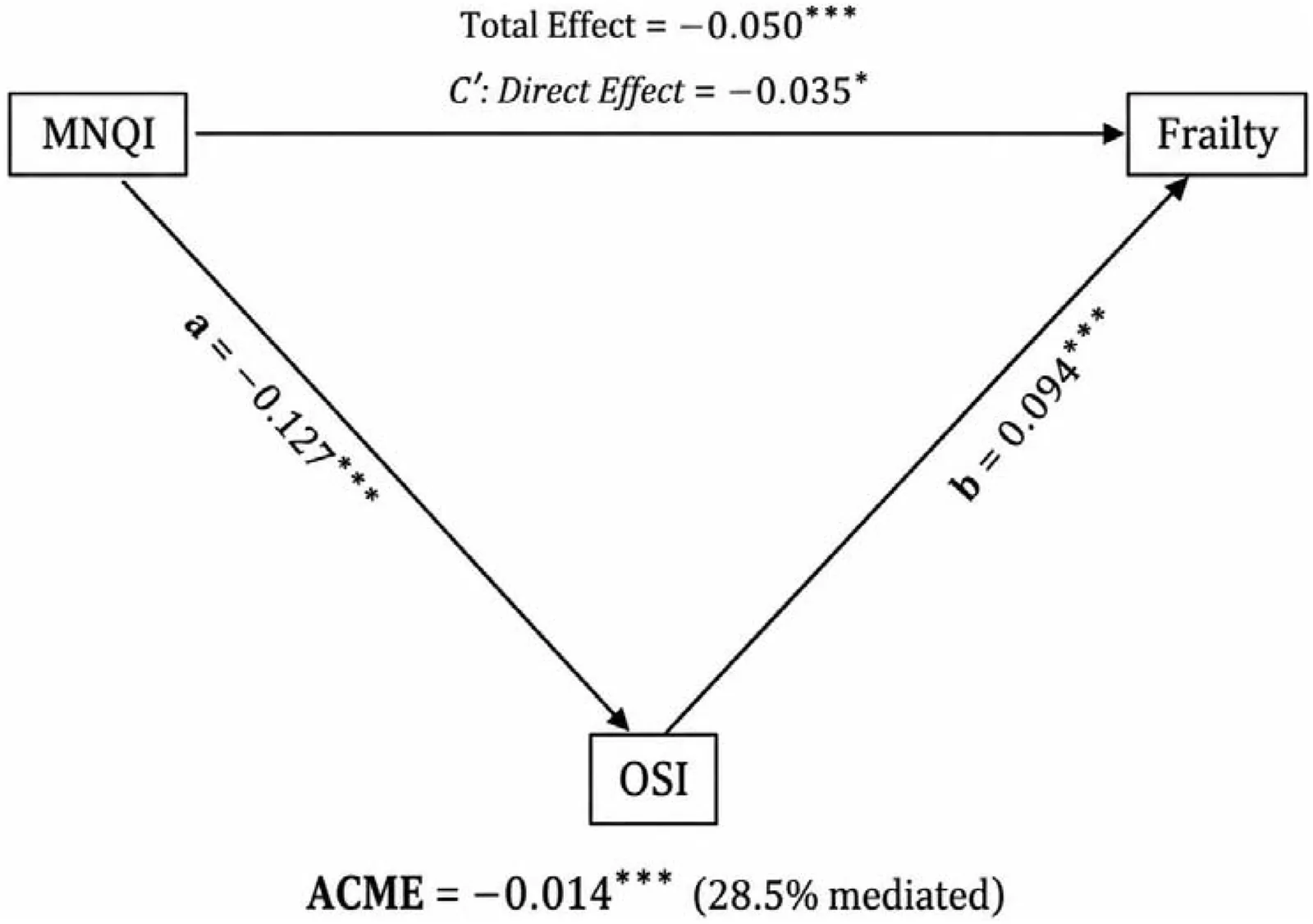
Mediation analysis of the association between the Methyl-Donor Nutrient Quality Index (MNQI) and frailty through Oxidative Stress Index (OSI) for the highest versus lowest MNQI tertile (T3 vs. T1), restricted to the frail versus robust comparison. The direct effect (ADE) represents the association between MNQI and frailty independent of OSI, whereas the indirect effect (ACME) represents the estimated effect through the OSI. The indirect effect through OSI accounted for approximately 28.5% of the total MNQI–frailty association. *P < 0.05; ***P < 0.001. ACME, average causal mediation effect; ADE, average direct effect; MNQI, Methyl-Donor Nutrient Quality Index; OSI, Oxidative Stress Index.

### 3.5 Sensitivity analysis

No significant interaction was observed between MNQI and HEI-2020 tertiles (P-interaction = 0.672), providing no evidence that the association between MNQI and frailty differed according to overall diet quality. In the sensitivity analysis restricted to adults aged ≥60 years, the inverse association between MNQI and frailty remained significant (Supplementary Table S4). In Model 3, compared with T1, the odds of frailty were lower for both T2 (OR = 0.70; 95% CI, 0.54–0.90; P = 0.006) and T3 (OR = 0.64; 95% CI, 0.48–0.86; P = 0.003), with a significant trend across MNQI categories (P for trend = 0.002). Associations with prefrailty were not statistically significant in Model 3.

## 4 Discussion

In this sample of U.S. middle-aged and older adults, higher dietary intake of methyl-donor nutrients, measured by the MNQI, was associated with lower odds of frailty in a dose-dependent manner. To our knowledge, this is the first study to examine the MNQI in relation to frailty. Higher oxidative stress was also associated with higher odds of prefrailty and frailty. Consistent with these findings, exploratory mediation analysis indicated an indirect effect of MNQI on frailty through oxidative stress, accounting for approximately 29% of the total association.

Moreover, the persistence of the MNQI–frailty association after adjustment for HEI suggests that methyl-donor nutrient adequacy captures aspects of diet relevant to frailty that are distinct from those reflected by overall diet quality. Additionally, when analyses were restricted to adults aged ≥60 years, the inverse association between MNQI and frailty remained significant, supporting the robustness of the findings in an older population in whom preserving physiological reserve and preventing frailty are particularly important for healthy aging and longevity.

The MNQI reflects the combined adequacy of interconnected nutrients involved in one-carbon metabolism. Higher dietary intake of methyl-donor nutrients has previously been associated with improved muscle strength, reduced phenotypic aging, and better cognitive function [22,33,34]. These outcomes represent important domains of health and function that may contribute to resilience during aging. Previous studies have also linked healthier dietary patterns and specific dietary components with lower frailty risk [[11], [12], [13], [14], [15], [16], [17],[23], [24], [25], [26], [27],^32^,[47], [48], [49], [50], [51], [52]]. The current findings extend this literature by supporting consideration of one-carbon metabolism–related nutrient adequacy when examining nutritional determinants of frailty.

The observed associations are biologically plausible given the central role of one-carbon metabolism in methylation reactions, genomic stability, cellular maintenance, and antioxidant defense [29,53]. Although methyl-donor nutrients are not classical antioxidants, they influence oxidative balance through several interconnected mechanisms, including regulation of antioxidant defense pathways, maintenance of mitochondrial function, homocysteine recycling, and support of S-adenosylmethionine synthesis for DNA methylation and epigenetic regulation [29,30,53,54]. Adequate methyl-donor availability sustains homocysteine metabolism and glutathione synthesis, thereby preserving antioxidant capacity, whereas inadequate intake may impair these processes and promote oxidative stress [21,44,55]. Nutrients such as folate, vitamins B6 and B12, choline, methionine, and zinc collectively support biological processes essential for maintaining physiological reserve and resilience with aging [21,29,30,[53], [54], [55], [56], [57], [58], [59]].

Oxidative stress has emerged as a key biological mechanism underlying frailty, contributing to cumulative cellular damage, chronic inflammation, mitochondrial dysfunction, and accelerated biological aging [19,47,56,57]. Excess reactive oxygen species relative to antioxidant defenses can impair cellular repair processes and disrupt metabolic homeostasis, ultimately eroding physiological reserve [19,56]. Consistent with this framework, the exploratory mediation analysis indicated a significant indirect effect through oxidative stress in the association between MNQI and frailty, aligning with prior NHANES-based studies reporting indirect effects through oxidative stress between dietary exposures and age-related outcomes [20,60]. The predominance of the direct effect, however, suggests that additional pathways may contribute, for example epigenetic regulation, mitochondrial function, and mechanisms involved in muscle maintenance and repair, all of which are influenced by methyl-donor nutrient availability [29,54,56,57].

Importantly, frailty is not necessarily irreversible, and growing evidence suggests that its progression may be delayed or mitigated through interventions targeting modifiable risk factors, including nutrition [[11], [12], [13], [14], [15], [16], [17]]. Our findings support the relevance of methyl-donor nutrient adequacy as one such target and highlight oxidative stress as a potential pathway linking diet to frailty that warrants further investigation. However, longitudinal and interventional studies are needed to confirm these relationships and establish causality.

Several limitations should be considered. First, the cross-sectional design precludes causal inference and does not allow the temporal direction of the observed associations among MNQI, oxidative stress, and frailty to be established. Reverse causality is also possible, as frailty may influence dietary intake and nutritional status rather than dietary intake solely influencing frailty. Second, although mediation analysis was used to explore oxidative stress as a potential pathway linking MNQI to frailty, the assumptions required for causal mediation, including appropriate temporal sequencing and the absence of unmeasured confounding, cannot be fully established in cross-sectional data. Accordingly, the mediation findings should be interpreted as exploratory and do not demonstrate a causal mechanism. Third, dietary intake was assessed using 24-h recalls, which are subject to recall bias and intra-individual variability. Additionally, due to NHANES survey design procedures, healthier participants were more likely to have insufficient data for frailty index construction and were excluded from the analysis. The generally more favorable health profiles among excluded participants suggest that this differential exclusion may have contributed to the relatively high prevalence of frailty in the analytic sample and introduced selection bias, potentially limiting the generalizability of the findings. Although methionine and betaine are important components of one-carbon metabolism, they were not available in the NHANES dietary nutrient files and therefore could not be included in the MNQI. Sampling weights were included as a covariate in the mediation analyses rather than applied as survey weights, which may limit the generalizability of these estimates. Finally, residual confounding by unmeasured factors cannot be excluded. Prospective studies are needed to establish temporal relationships among methyl-donor nutrient adequacy, oxidative stress, and frailty, while intervention studies are warranted to determine whether improving methyl-donor nutrient adequacy can prevent or mitigate frailty.

Despite these limitations, this study has several notable strengths, including the use of a large U.S. population-based sample, a comprehensive 53-item frailty index capturing multiple physiological domains, and the integration of dietary, biomarker, and mediation analyses within a unified framework. The MNQI represents a novel composite index reflecting the synergistic roles of methyl-donor nutrients within one-carbon metabolism, and findings remained robust across sensitivity analyses and mediation models with diet quality adjustment.

In conclusion, higher dietary intake of methyl-donor nutrients was associated with lower odds of frailty in a large U.S. population-based sample of middle-aged and older adults, with oxidative stress potentially representing a modest pathway linking methyl-donor nutrient quality and frailty. The independence of this association from overall diet quality supports the relevance of one-carbon metabolism nutrients as a potential modifiable nutritional target for preserving physiological reserve, reducing frailty risk, and supporting healthy aging and longevity. Longitudinal studies and randomized clinical trials are needed to establish causality and inform evidence-based dietary recommendations.

## Author contributions

GB designed the research; GB conducted the research and analyzed the data under the supervision of JDS; GB and HS wrote the paper; GB had primary responsibility for final content. All authors (GB, HS, ASK, SMJ, ASR, and JDS) reviewed, edited, and approved the final manuscript.

## Declaration of Generative AI and AI-assisted technologies in the writing process

During the preparation of this work, the authors used ChatGPT (OpenAI) and Claude (Anthropic) to assist with language editing and manuscript organization. After using these tools, the authors reviewed and edited the content as needed and take full responsibility for the content of the publication.

## Funding

This work was supported by the National Institute on Aging of the National Institutes of Health (grant number K01AG078545 to GB). The funding source had no role in the study design; collection, analysis, or interpretation of data; writing of the report; or the decision to submit for publication.

## Data availability

Data are publicly available from NHANES through the National Center for Health Statistics at https://www.cdc.gov/nchs/nhanes/ (accessed April 15, 2026). Analytic code is available from the corresponding author upon reasonable request.

## Declaration of competing interest

All authors declare no conflicts of interest.
