# Supplementary material for: Dietary intake of methyl-donor nutrients is associated with frailty and partially mediated by oxidative stress in U.S. middle-aged and older adults
Source: J Nutr Health Aging. 2026 Nov 4;31(1):101025. doi: 10.1016/j.jnha.2026.101025 (PMC13756254; doi:10.1016/j.jnha.2026.101025)
Supplement: Supplementary file 1 [file mmc1.docx]

**Supplements**

26,282 participants
aged ≥40 years
NHANES 2005–2018

Excluded: n = 10,909
(<80% Frailty Index items completed) *

15,373 participants included

Excluded: n = 1,618
• Missing MNQI: n = 934
• Missing OSI: n = 684

13,755 participants included

Excluded: n = 227
(Missing covariates)

Final analytic sample:
n = 13,528

**FIGURE S1.** Flow chart of the screening process for the selection of the study population.

* Participants who reported no limitations in work ability (PFQ049), work capacity (PFQ051), mobility (PFQ054), or cognition (PFQ057) were not administered the physical functioning module (PFQ061) **by design**. As PFQ061 contributed substantially to the frailty index, some healthier participants had insufficient data (<80% of items completed) and were excluded from the analysis

| **SUPPLEMENTARY TABLE S1 Variables in the Frailty Index and Their Respective Scores** | | |
| --- | --- | --- |
| **Item** | **Scoring** | **NHANES Variable** |
| **Cognition** | | |
| 1. Experience confusion or memory problems | Yes = 1, No = 0 | PFQ057 |
| **Functional Limitations*** | | |
| 2. Managing money difficulty | No difficulty = 0, Some difficulty = 0.33, Much difficulty = 0.66, Unable to do = 1 ("Do not do this activity" coded as missing) | PFQ061A |
| 3. Walking a quarter mile difficulty |  | PFQ061B |
| 4. Walking up ten steps difficulty |  | PFQ061C |
| 5. Stooping, crouching, or kneeling difficulty |  | PFQ061D |
| 6. Lifting or carrying difficulty |  | PFQ061E |
| 7. Doing household chores difficulty |  | PFQ061F |
| 8. Preparing meals difficulty |  | PFQ061G |
| 9. Walking between rooms on same floor |  | PFQ061H |
| 10. Standing up from armless chair difficulty |  | PFQ061I |
| 11. Getting in and out of bed difficulty |  | PFQ061J |
| 12. Using fork, knife, or cup difficulty |  | PFQ061K |
| 13. Dressing yourself difficulty |  | PFQ061L |
| 14. Standing for long periods difficulty |  | PFQ061M |
| 15. Sitting for long periods difficulty |  | PFQ061N |
| 16. Reaching up overhead |  | PFQ061O |
| 17. Grasping or holding small objects difficulty | | PFQ061P |
| 18. Going out to movies or events |  | PFQ061Q |
| 19. Attending social events difficulty |  | PFQ061R |
| 20. Leisure activity at home difficulty |  | PFQ061S |
| 21. Pushing or pulling large objects difficulty |  | PFQ061T |
| **Depressive Symptoms** | | |
| 22. Little interest in doing things | Nearly every day = 1, More than half the days = 0.66, Several days = 0.33, Not at all = 0 | DPQ010 |
| 23. Feeling down, depressed, or hopeless |  | DPQ020 |
| 24. Trouble sleeping or sleeping too much |  | DPQ030 |
| 25. Feeling tired or having little energy |  | DPQ040 |
| 26. Poor appetite or overeating |  | DPQ050 |
| 27. Feeling bad about yourself |  | DPQ060 |
| 28. Trouble concentrating on things |  | DPQ070 |
| **Comorbidities** | | |
| 29. Doctor ever told you had arthritis | Yes = 1, No = 0 | MCQ160A |
| 30. Ever told you had a thyroid problem | Yes = 1, No = 0 | MCQ160M |
| 31. Ever told you had chronic bronchitis | Yes = 1, No = 0 | MCQ160K |
| 32. Ever told you had cancer or malignancy | Yes = 1, No = 0 | MCQ220 |
| 33. Ever told you had congestive heart failure | Yes = 1, No = 0 | MCQ160B |
| 34. Ever told you had coronary heart disease | Yes = 1, No = 0 | MCQ160C |
| 35. Ever told you had angina pectoris | Yes = 1, No = 0 | MCQ160D |
| 36. Ever told you had a heart attack | Yes = 1, No = 0 | MCQ160E |
| 37. Ever told you had a stroke | Yes = 1, No = 0 | MCQ160F |
| 38. Ever told you had high blood pressure | Yes = 1, No = 0 | BPQ020 |
| 39. Doctor told you have diabetes | Yes = 1, Borderline = 0.5, No = 0 | DIQ010 |
| 40. Ever told you had weak or failing kidneys | Yes = 1, No = 0 | KIQ022 |
| 41. Urine leakage bothers you | Greatly = 1, Very much = 0.75, Somewhat = 0.5, Only a little = 0.25, Not at all = 0 | KIQ050 |
| **Healthcare Utilization and Self-Reported Health** | | |
| 42. General health condition | Excellent, Very good, Good = 0; Fair, Poor = 1 | HUQ010 |
| 43. Health now compared with 1 year ago | Worse = 1, Better or same = 0 | HUQ020 |
| 44. Overnight hospital patient in last year | Yes = 1, No = 0 | HUQ071 |
| 45. Times received healthcare over past year | None = 0; 1–4 times = 0.5; ≥5 times = 1 | HUQ051 |
| 46. Number of prescription medicines taken | None = 0; 1–4 = 0.5; ≥5 = 1 | RXDCOUNT |
| **Physical Anthropometry** | | |
| 47. Body mass index (kg/m²) | <18.5 or ≥30 = 1; 25–29.9 = 0.5; 18.5–24.9 = 0 | BMXBMI |
| **Laboratory Values** | | |
| 48. Glycohemoglobin (%) | ≤5.7% = 0; >5.7% = 1 | LBXGH |
| 49. Red blood cell count (million cells/µL) | Male: ≥4.7 and <6.1 = 0, other = 1; Female: ≥4.2 and <5.4 = 0, other = 1 | LBXRBCSI |
| 50. Hemoglobin (g/dL) | Male: ≥13.5 and <18 = 0, other = 1; Female: ≥12 and <16 = 0, other = 1 | LBXHGB |
| 51. Red cell distribution width (%) | ≥11.6 and <14.6 = 0; other = 1 | LBXRDW |
| 52. Lymphocyte percent (%) | ≥20 and <40 = 0; other = 1 | LBXLYPCT |
| 53. Segmented neutrophils percent (%) | ≥40 and <80 = 0; other = 1 | LBXNEPCT |
| All variables were coded such that higher scores indicate worse health status. The frailty index (FI) was calculated as the sum of deficit scores divided by the total number of non-missing items (range: 0–1). Participants with data on at least 80% of items (≥43 of 53) were included. FI categories: robust (FI <0.10), prefrail (FI 0.10–0.24), frail (FI ≥0.25).  FI, frailty index; NHANES, National Health and Nutrition Examination Survey.  *Items 2–21 (PFQ061A–T) were administered only to participants who reported at least one limitation in work ability (PFQ049), work capacity (PFQ051), mobility (PFQ054), or cognition (PFQ057), or who reported any activity limitation (PFQ059). Participants aged ≤59 years who denied all limitations were routed away from this module by survey design. Missing responses due to this skip pattern were treated as missing in the frailty index calculation; participants with data on fewer than 80% of items (< 43 of 53) were excluded from analyses | | |

| **SUPPLEMENTARY TABLE S2. Components and Scoring Criteria for the Methyl-Donor Nutrient Quality Index (MNQI)** | | | |
| --- | --- | --- | --- |
| **Nutrient** | **Lower cut-point (adequacy threshold)** | **Upper bound (tolerable upper intake level)** | **Score (points)** |
| Protein | ≥ ⅔ of 0.8 g/kg/day | ≤ 2.0 g/kg/day | 2 |
| Folate | ≥ ⅔ of 400 µg/day | ≤ 1,000 µg/day | 2 |
| Choline, men | ≥ ⅔ of 550 mg/day | ≤ 3,500 mg/day | 2 |
| Choline, women | ≥ ⅔ of 425 mg/day | ≤ 3,500 mg/day | 2 |
| Riboflavin, men | ≥ ⅔ of 1.3 mg/day | Not applied | 1 |
| Riboflavin, women | ≥ ⅔ of 1.1 mg/day | Not applied | 1 |
| Vitamin B6, age 19–50 y | ≥ ⅔ of 1.3 mg/day | ≤ 100 mg/day | 1 |
| Vitamin B6, men ≥51 y | ≥ ⅔ of 1.7 mg/day | ≤ 100 mg/day | 1 |
| Vitamin B6, women ≥51 y | ≥ ⅔ of 1.5 mg/day | ≤ 100 mg/day | 1 |
| Vitamin B12 | ≥ ⅔ of 2.4 µg/day | Not applied | 1 |
| Zinc, men | ≥ ⅔ of 11 mg/day | ≤ 40 mg/day | 1 |
| Zinc, women | ≥ ⅔ of 8 mg/day | ≤ 40 mg/day | 1 |
| The total MNQI score ranges from 0 to 10; higher scores indicate greater methyl-donor nutrient adequacy. Nutrients assigned 2 points (protein, folate, choline) serve as primary methyl-group donors; nutrients assigned 1 point (riboflavin, vitamin B6, vitamin B12, zinc) are essential cofactors supporting one-carbon metabolism.  Nutrient intake was calculated as the mean of Day 1 and Day 2 dietary recalls when both were available; if only one recall was available, Day 1 intake was used. Participants received the assigned score if intake met the adequacy criterion (i.e., ≥ lower cut-point and ≤ upper bound where applicable) and 0 otherwise.  MNQI, Methyl-Donor Nutrient Quality Index; UL, tolerable upper intake level. | | | |

| **SUPPLEMENTARY TABLE S3. Baseline Characteristics by Data Completeness Status** | | | | |
| --- | --- | --- | --- | --- |
| **Variable** | **Category** | **Missing N (%)** | **Complete N (%)** | **P value** |
| **Sex** | Male | 6,120 (48.1%) | 6,686 (45.9%) | 0.051 |
|  | Female | 6,643 (51.9%) | 6,842 (54.1%) |  |
| **Race/Ethnicity** | Non-Hispanic White | 4,711 (67.3%) | 6,793 (76.1%) | <0.001 |
|  | Hispanic | 3,344 (13.2%) | 2,914 (8.6%) |  |
|  | Non-Hispanic Black | 2,941 (11.7%) | 2,823 (9.7%) |  |
|  | Asian/Other | 1,767 (7.8%) | 998 (5.6%) |  |
| **Education** | Less than high school | 3,385 (13.8%) | 3,979 (18.3%) | <0.001 |
|  | High school/equivalent | 2,743 (22.0%) | 3,291 (25.9%) |  |
|  | Above high school | 6,584 (64.2%) | 6,258 (55.9%) |  |
| **Marital Status** | Married/living with partner | 8,238 (71.7%) | 7,796 (63.3%) | <0.001 |
|  | Living alone | 4,496 (28.3%) | 5,732 (36.7%) |  |
| **Smoking Status** | Never | 7,309 (57.2%) | 6,332 (47.1%) | <0.001 |
|  | Former | 3,012 (24.6%) | 4,787 (35.6%) |  |
|  | Current | 2,417 (18.2%) | 2,400 (17.3%) |  |
| **Alcohol use,^1^ n (%)** | Never | 1,405 (9.2%) | 2,068 (12.3%) | <0.001 |
|  | Former | 1,864 (15.0%) | 3,827 (25.0%) |  |
|  | Current | 6,617 (75.8%) | 7,470 (62.7%) |  |
| **Diet quality (HEI),^2^ mean ± SD** | | 54.8 ± 12.2 | 55.5 ± 13.3 | 0.029 |
| **BMI,^3^ kg/m², mean ± SD** |  | 29.0 ± 6.0 | 29.8 ± 6.9 | <0.001 |
| **Total energy intake, kcal/day, mean ± SD** | | 2,113 ± 756 | 1,920 ± 733 | <0.001 |
| **Chronic disease burden, n (%)** | None | 6,589 (56.8%) | 3,061 (22.2%) | <0.001 |
|  | One condition | 3,766 (29.6%) | 4,408 (34.1%) |  |
|  | Two or more conditions | 2,408 (13.6%) | 6,059 (43.7%) |  |
| **MNQI,^4^ n (%)** | T1 (score 0–7) | 3,364 (29.7%) | 5,418 (35.3%) | <0.001 |
|  | T2 (score 8) | 3,120 (33.1%) | 4,218 (32.5%) |  |
|  | T3 (score 9–10) | 3,303 (37.2%) | 3,892 (32.2%) |  |
| **Oxidative stress index,^5^ mean ± SD** | | −0.16 ± 0.85 | 0.02 ± 0.96 | <0.001 |
| Values are n (weighted %) or mean ± SD. P values were derived from survey-weighted linear regression for continuous variables and survey-weighted χ² tests for categorical variables.  1 There were 163 missing values for alcohol use.  2 HEI, Healthy Eating Index.  3 BMI, body mass index.  4 MNQI, Methyl-Donor Nutrient Quality Index (range 0–10; higher scores indicate greater methyl-donor nutrient adequacy); tertiles defined as T1 (0–7), T2 (8), and T3 (9–10).  5 Oxidative stress index represents a standardized composite z-score; higher values indicate greater oxidative stress. | | | | |

| **SUPPLEMENTARY TABLE S4.** Survey-Weighted Association between Methyl-Donor Nutrient Quality and Frailty Among Older Adults Aged ≥60 Years | | | | | | |
| --- | --- | --- | --- | --- | --- | --- |
|  | **Model 1** | | **Model 2** | | **Model 3** | |
| **MNQI** | OR  (95% CI) | P value | OR  (95% CI) | P value | OR  (95% CI) | P value |
| **Prefrail vs. Robust** | | | | | | |
| T2 vs T1 | 0.81 (0.66–0.99) | 0.044 | 0.86 (0.70–1.05) | 0.136 | 0.96 (0.78–1.18) | 0.693 |
| T3 vs T1 | 0.74 (0.60–0.93) | 0.009 | 0.85 (0.68–1.06) | 0.157 | 0.83 (0.64–1.09) | 0.175 |
| P for trend | 0.009 | | 0.170 | | 0.169 | |
| **Frail vs. Robust** | | | | | | |
| T2 vs T1 | 0.47 (0.36–0.60) | <0.001 | 0.55 (0.43–0.71) | <0.001 | 0.70 (0.54–0.90) | 0.006 |
| T3 vs T1 | 0.45 (0.35–0.57) | <0.001 | 0.60 (0.47–0.75) | <0.001 | 0.64 (0.48–0.86) | 0.003 |
| P for trend | <0.001 | | <0.001 | | 0.002 | |

Survey-weighted multinomial logistic regression. Robust was the reference category.

Model 1: Unadjusted.

Model 2: Adjusted for age, sex, race/ethnicity, education level, and marital status.

Model 3: Adjusted for the variables in Model 2 + smoking status, alcohol use, energy intake, diet quality (HEI), and BMI.

MNQI, Methyl-Donor Nutrient Quality Index; OR, odds ratio; HEI, Healthy Eating Index; BMI, body mass index.
